# Intra operative lesion of the pelvic ureter solved in a minimally invasive manner

**Published:** 2014-09-25

**Authors:** RA Stoica, T Enache, N Iordache

**Affiliations:** * "Sf. Ioan" Clinical Emergency Hospital, General Surgery Department; ** "Panait Sarbu" Clinical Obstetrics and Gynaecology Hospital

**Keywords:** laparoscopy, hysterectomy, ureter lesion

## Abstract

Abstract

Ureteral lesions during open hysterectomy, vaginal hysterectomy or laparoscopic hysterectomy have a rate of 0.2% up to 6%. Multiple complications may occur if the lesion is not recognised intra operatively: hydronephrosis, anuria (bilateral lesion), ureterovaginal fistula, ileus, peritonitis. The rate of recognition of an intra operative ureter lesion is 30% and it could rise up to 90% when cystoscopy with ureteroscopy is used at the end of the intervention.

The article presents the case of a 46-year-old patient with uterine fibromatosis, whose pelvic ureter was sectioned during surgery. The lesion was recognised during surgery because, at the end of each intervention, the diuresis was stimulated by injecting Furosemide in order to detect the lesions of the ureters and urinary bladder.

## Introduction

Hysterectomy is the most common gynaecological intervention. The ureter lesions during classic, vaginal or laparoscopic hysterectomy vary between 0.2% and 6% [**1-5**] . The causes that lead to this type of lesions are: neoplasia, radiotherapy, endometriosis, the cervix fibromatosis or the fibroids within ligaments. The most common regions where ureters can be sectioned are: when we approach the suspensory ligament of the ovary, the uterosacral ligament and uterine artery. The recognition and the treatment of these lesions during surgery lead to a lower morbidity rate. Unfortunately, 70% of the lesions [**6**] are not recognised during surgery and they are discovered later through hydronephrosis, anuria (bilateral lesion), watery vaginal leaks (urine), paralytic ileus or even peritonitis. 

 The presentation of the case: The patient, B.V., 46 years old, diagnosed with moderate anaemia, abnormal gynaecologic haemorrhage, cervical myoma, in a Gynaecologic clinic, where the surgical intervention proposed (laparotomy, total hysterectomy with bilateral salpingo-oophorectomy) could not be performed, the patient could not be intubated because of the low visibility of the oropharynx (score IV Mallampati - only the hard palate was visible), was transferred and hospitalized in the General Surgery Unit for the procedure, this time laparoscopic. The tracheal intubation was made with an Eschmann tube and, as a backup plan, in case of failure, a bronchoscope was prepared. 

 After achieving pneumoperitoneum, during exploration, an intense pelvic adhesion syndrome, posterior to the uterus, could be seen, which involved ovaries, salpinges, uterus and rectum. After a laboriously adhesiolysis, the intervention started. What should be highlighted is the importance of the uterine manipulator during laparoscopic hysterectomies, the intervention being a lot easier when the uterus is well manipulated and brought abdominally, being aggravated or even impossible when, due to different reasons, the uterine manipulator cannot be inserted. In this case, the manipulator could not be fixed properly because of the cervical myoma which does not allow the insertion of the device inside the uterus and so, the exposure is deficient. 

**Fig. 1:**
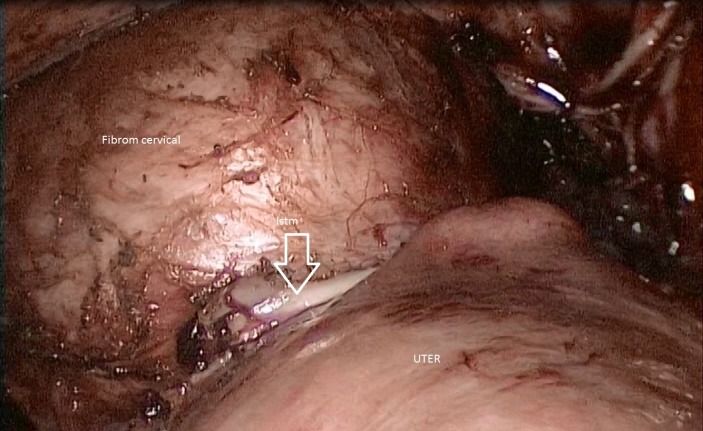
Dissection of urinary bladder from the cervical myoma and the mobilization of the fibroid

**Fig. 2:**
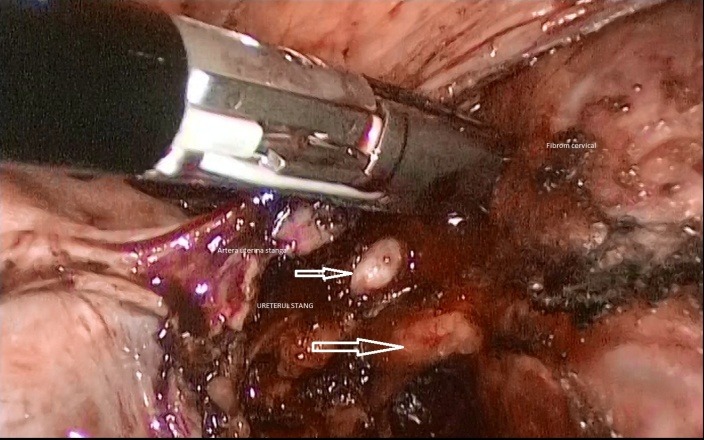
Left uterine artery

**Fig. 3:**
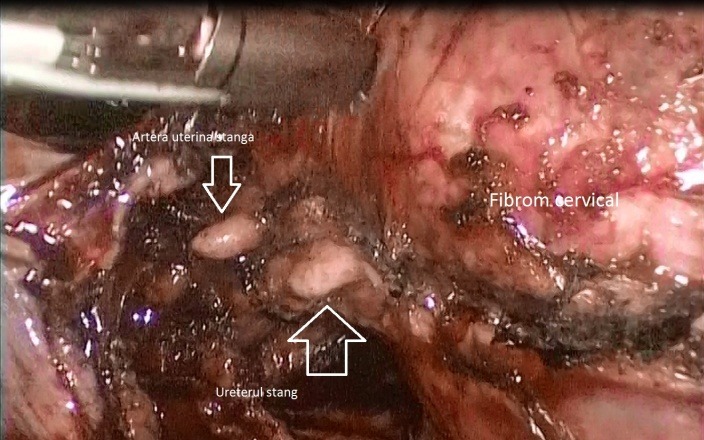
The ureter was mistaken for the uterine artery

**Fig. 4:**
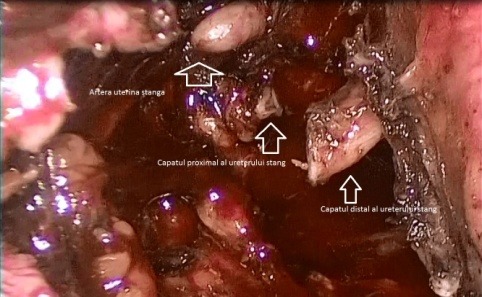
LigaSure was used to seal the blood vessels but it did not also seal the ureter

 After the dissection of urinary bladder from the cervical myoma and the mobilization of the fibroid (**Fig. 1**), while approaching the left uterine artery (**Fig. 2**), because of the lateral growth of the myoma, which had an intimate contact with the ureter, the ureter was mistaken for the uterine artery (**Fig. 3**). The lesion made from the beginning was not realised, even if the LigaSure was used to seal the blood vessels, it did not also seal the ureter (**Fig. 4**).

**Fig. 5:**
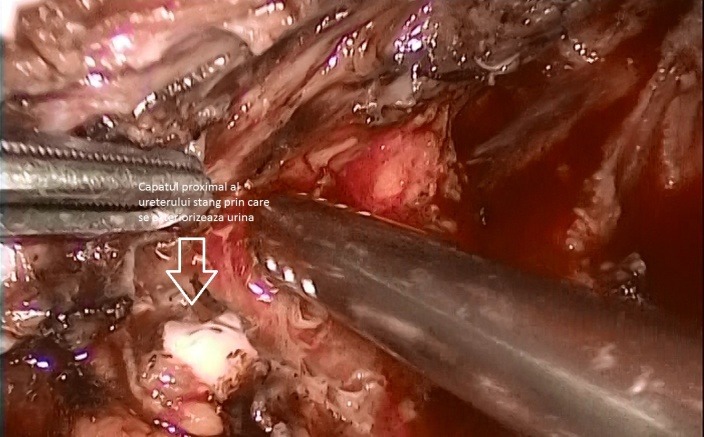
LigaSure was used to seal the blood vessels but it did not also seal the ureter

**Fig. 6:**
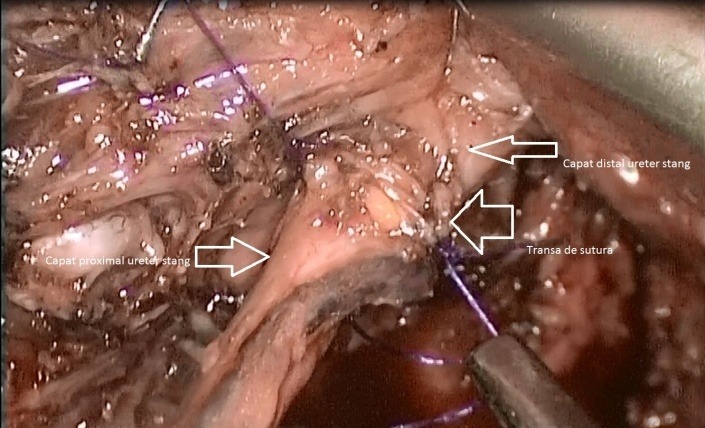
LigaSure was used to seal the blood vessels but it did not also seal the ureter

**Fig. 7:**
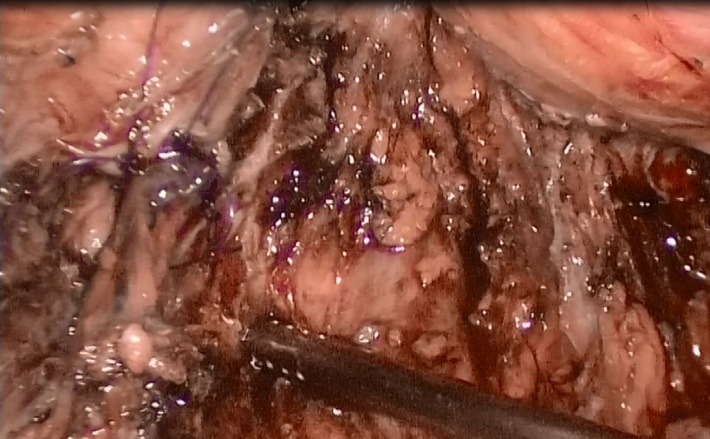
LigaSure was used to seal the blood vessels but it did not also seal the ureter

 After completing the hysterectomy, extracting the uterus, ovaries and salpinges through the vagina, sealing it, after injecting Furosemide, the presence of a liquid in the pelvic area and a big quantity of "ascites" were noticed. After a thoroughly inspection, the proximal end of the left ureter, whereby urine was leaking discontinuously was discovered (**Fig. 5**). A trial, without success however, was done to visualise the distal end. Help was asked for from the colleagues in the Urology Unit, who performed a cystoscopy with a catheterisation of both ureters. The exploration certified the integrity of the right ureter and during the catheterisation of the left one, the catheter appeared in the abdominal cavity. The guidance stitch was led in the proximal end of left ureter and a double JJ tube was fixed. Guided by the double JJ tube, a head to head suture of the ureters’ ends with 4 suture points with swaged 4-0 absorbable in a laparoscopic manner was realized (**Fig. 6**). At the end of the intervention the leak free was checked and 2 drainage tubes were left in the pelvis (**Fig. 7**). 

 The Foley catheter was maintained for 7 days. The JJ catheter was maintained for 3 months, at first in order to assure a good drainage of the ureter and to lower the tension within the anastomosis and then to prevent the stenosis of the ureter. 

## Result

The patient was discharged after 10 days, without fever, without abdominal collections at ultrasound and with the presence of intestinal transit for gas and faeces. 

 After 3 months, the JJ catheter was cystoscopically extracted and at 9 months from surgery the patient was asymptomatic, the ultrasound of the abdomen revealed both kidneys with a normal aspect, without hydronephrosis or abdominal collections. 

## Conclusions 

The early discovery of the ureter lesions is the ideal solution to solve these types of intra operative incidents, this way avoiding an important morbidity rate. 

 The administration of Furosemide at the end of the surgery is considered an efficient and cheap method of recognising these lesions, compared with cystoscopy, the recommended alternative of detecting this type of lesions in literature. 

 The recognition of the lesion during surgery led to a simple and elegant way of solving the ureter lesion, through a minimally invasive approach. 
